# A Study on the Influence of Process Parameters on the Workpiece Surface Quality in the Cutting of Hard and Brittle Materials with Trepanning Drill

**DOI:** 10.3390/ma16186114

**Published:** 2023-09-07

**Authors:** Ruijiang Yu, Shujuan Li, Zhengkang Zou, Lie Liang, Jiawei Zhang

**Affiliations:** School of Mechanical and Precision Instrument Engineering, Xi’an University of Technology, Xi’an 710048, China; 1170211009@stu.xaut.edu.cn (R.Y.); 1170211011@stu.xaut.edu.cn (L.L.);

**Keywords:** surface roughness, exit-chipping width, trepanning machining, crack

## Abstract

Hard and brittle materials have excellent physical and mechanical properties and are widely used in the fields of microelectronics and optoelectronics. However, due to their high hardness and brittleness, the machining quality of a workpiece struggles to meet the requirements of practical applications. In order to improve the surface quality of deep-hole machining of hard and brittle materials, this article analyzes the formation mechanism of surface roughness and the exit-chipping width during the drilling machining of hard and brittle materials and establishes a mathematical prediction model for the surface roughness and the exit-chipping width of hard and brittle materials using a trepanning cutter. The experimental study on K9 optical glass machining shows that the surface roughness of the workpiece and the exit-chipping width increase with the increase in feed rate, and decrease with the increase in rotational speed. Through comparison and verification between theoretical and experimental values, the average errors of workpiece surface roughness and the exit-chipping width are 13.15% and 6.73%, respectively. This article analyzes the reasons for the errors. The results indicate that the theoretical model proposed in this article can be used to predict the surface roughness and the exit-chipping width of hard and brittle materials processed under the same conditions, providing a theoretical basis to optimize process parameters to improve the surface quality of the workpiece.

## 1. Introduction

Hard and brittle materials have been widely used in aerospace, electronic equipment, and other fields due to their unique material properties, such as high hardness, high strength, low density, and good chemical stability [1,2,3,4]. However, these materials are typically difficult-to-machine materials due to their high hardness, high brittleness, and low fracture toughness. It is quite difficult to achieve high precision, efficient, and high-surface-quality machining of such materials, especially high surface quality, which has a significant impact on the performance of parts and assembly positioning accuracy and seriously restricts the wider application of hard and brittle materials [5,6,7]. Therefore, it is necessary to conduct more in-depth research and improve on the machining mechanism of hard and brittle materials and the formation mechanism of workpiece machining surface quality.

For drilling hard and brittle materials, the surface roughness of the hole and the exit-chipping size are important indicators for evaluating the quality of hole machining. The surface roughness of workpieces has a significant impact on their working strength, accuracy, corrosion resistance, and compatibility, and is a widely considered focus in manufacturing processes. Meanwhile, the surface roughness of the workpiece directly reflects the material removal method and forming mechanism. The exit-chipping size directly reflects the integrity of the hole body, and it has a significant impact on the performance of the parts and assembly positioning accuracy. Therefore, it is necessary to evaluate the surface roughness of the processed hole and the exit-chipping size, as well as study the influencing factors. At present, on the basis of extensive research on the removal mechanism of hard and brittle materials, progress has been made in the formation mechanism of surface quality of machined hard and brittle materials. Ding et al. [8] conducted rotary ultrasonic machining (RUM) and conventional drilling (CD) experiments using a diamond trepanning drill. The effects of two technologies on axial force, torque, hole quality, and drilling surface roughness were compared. The research results indicate that the surface roughness of the drilling holes obtained using RUM is lower than CD. Zheng et al. [9] conducted constant feed rate drilling experiments on Al_2_O_3_ and S_i_C materials using a diamond drill and studied the changes in the axial force and surface microstructure of the bore wall during the drilling machining. Gao et al. [10] analyzed the effects of axial force and rotational speed on drilling efficiency and hole quality by conducting experiments on machining Al_2_O_3_ armored ceramics using a fixed diamond drill bit. The experimental results indicate that increasing axial force and rotational speed can improve drilling efficiency, but excessive axial force and excessive rotational speed can also reduce drilling efficiency. Zheng et al. [11] studied the effects of process parameters such as rotational speed and axial force on machining efficiency and hole wall surface quality by conducting experiments on drilling ceramic composite armor with a fixed diamond thin-walled trepanning cutter. Li et al. [12,13] conducted experiments on the rotary ultrasonic constant feed rate drilling of hard and brittle materials, and studied the effects of parameters such as rotational speed, feed rate, and ultrasonic power on cutting force and exit-chipping size after machining. Abdallah et al. [14] conducted machining experiments on soda glass using a fixed diamond abrasive cutter and studied the effects of cutter surface abrasive density, rotational speed, and feed rate of a workpiece on the axial force and exit-chipping situation. The study found that low rotational speed and high feed rate would reduce the exit-chipping size width. Sharma et al. [5] studied the effects of drilling force and cutting temperature on the surface integrity of holes by drilling optical glass. The study showed that reducing cutting force and drilling temperature can reduce the stress during drilling, thereby improving the surface integrity of the workpiece. Lv et al. [6] explored the formation mechanism of exit-chipping during rotary ultrasonic drilling (RUD) and conventional drilling (CD) of BK7 glass from both theoretical and experimental perspectives. They proposed a quantitative relationship between instantaneous extrusion pressure and crack propagation direction that is inversely proportional and discussed the influence of the expansion direction of initial and annular cracks on the formation mechanism of exit-chipping. Wang et al. [15] established a prediction model for the hole-chipping size during the rotary ultrasonic drilling of hard and brittle materials, taking into account the influence of cutting force and machining-induced subsurface crack damage on hole-chipping. Table 1 compares previous research on the trepanning processing of hard and brittle materials.

The above achievements have conducted multiple studies on the surface quality and influencing factors of hard and brittle materials from experimental and theoretical perspectives, providing an important basis for the high-surface-quality machining of hard and brittle materials. However, these studies mainly analyze the factors that affect the surface quality of a workpiece through experiments and do not provide a complete theoretical analysis of the surface roughness of a workpiece and the exit-chipping width in the machining of hard and brittle materials with the trepanning cutter. At the same time, they do not analyze the trend of the influence of process parameters on the surface quality of machining. Therefore, it is necessary to develop a theoretical model that accurately predicts the surface roughness of the workpiece and the exit-chipping width, including process parameters, to provide an operable theoretical basis for improving the surface quality of the workpiece.

This article proposes a mathematical prediction model for the surface roughness and exit-chipping width of hard and brittle materials processed with a fixed abrasive trepanning cutter by analyzing the formation mechanism of workpiece surface roughness and exit-chipping width during the drilling process of hard and brittle materials. This model theoretically analyzes the main factors that affect the surface quality of workpieces, providing a predictable theoretical basis for improving the degree of surface quality of workpieces. Based on this theoretical model, optimizing process parameters at different times can achieve a perfect balance between workpiece surface quality and machining efficiency. By conducting machining experiments on optical glass (K9), this article analyzes the effects of different process parameters on the surface roughness of the workpiece and the exit-chipping width and verifies the effectiveness and rationality of the established mathematical prediction model. The research results can provide a theoretical basis for the machining of hard and brittle materials with similar material properties, such as silicon, ceramics, silicon carbide, etc.

## 2. Materials and Methods

### 2.1. The Formation Mechanism of Surface Roughness of Workpiece

**Figure 1:**
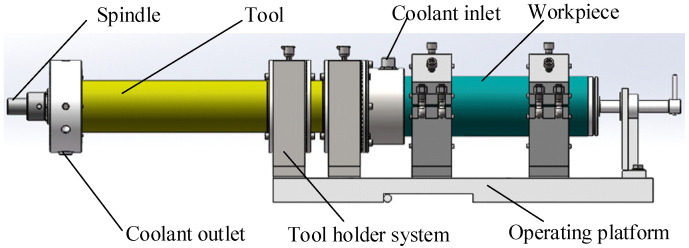
The schematic of trepanning machining.

Fixed abrasive trepanning machining is a composite machining technology that combines the material removal methods of traditional deep-hole drilling and grinding. Figure 1 shows the schematic diagram of fixed abrasive trepanning machining. The cutter with diamond particles is feeding towards the workpiece at a constant speed or pressure while rotating at high speed.

**Figure 2:**
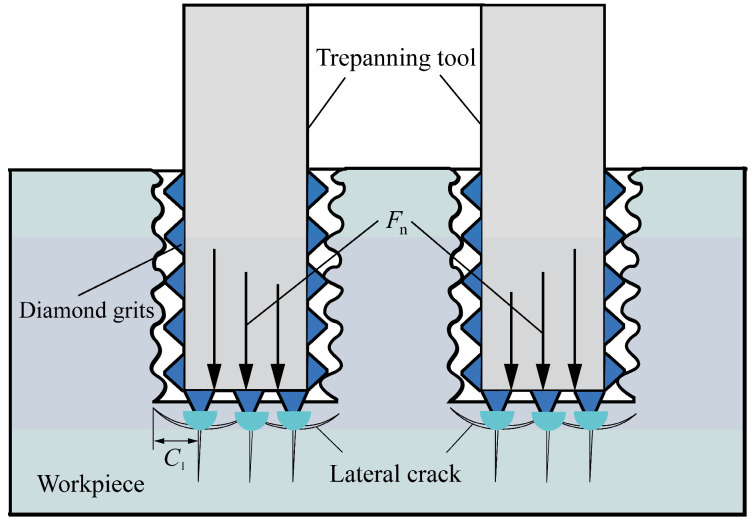
Formation mechanism of surface roughness of hole wall.

In the formation process of hard and brittle material hole surfaces, the role played by the end face abrasive particles and the side abrasive particles of the cutter is not the same. The removal of materials is mainly completed by the abrasive particles on the end face of the cutter, so the formation of the hole wall surface is mainly influenced by the removal mechanism of the abrasive material on the end face. The abrasive particles on the side of the cutter only serve to trim and improve the workpiece. The diamond abrasive particles on the end face of the cutter press into the surface of the workpiece like small indenters, generating median/radial cracks and lateral cracks inside the workpiece. The formation and propagation of lateral cracks cause the material to be removed from the surface in the form of brittle fracture, while the lateral cracks generated by the abrasive particles on the outermost side of the cutter end face extend into the hole wall of the workpiece. As the cracks in the hole wall continue to increase, the material wrapped by the lateral cracks falls off as a whole under the action of the lateral abrasive particles, forming an uneven hole wall surface. The formation mechanism is shown in Figure 2. In Figure 2, the roughness of the hole wall surface is mainly affected by the length of lateral cracks, and the length of lateral cracks is mainly affected by the axial force acting on a single abrasive particle. The peak and deep valley on the micro profile of the hole wall surface form the surface roughness of the workpiece, and the maximum distance between the peak and deep valley is equal to the maximum length of the transverse crack.

According to the theory of indentation fracture mechanics, the lateral crack length *C_l_* generated by a single abrasive particle pressing into a brittle material can be calculated using the following equation [16]:

(1) $$C_{l}=\alpha {\left(\cot\varphi \right)}^{5/12}\frac{E^{3/8}}{H_{v}^{1/2}K_{\mathrm{IC}}^{1/2}}F_{\mathrm{an}}^{5/8}$$

where *α* is a dimensionless constant, independent of the material/head system, taken as 0.226 [17]; *φ* is the half angle of the abrasive tip (°); *E* is the elastic modulus of the material (GPa); *K*_IC_ is the fracture toughness of the material (MPa·m^1/2^); *H*_v_ is the hardness of the material (GPa); and *F*_an_ is the axial force exerted on a single abrasive particle (N).

The calculation formula for the axial force *F*_n_ exerted on the cutter during the trepanning machining is expressed as follows [18]:

(2) $$F_{n}=\frac{15\pi \cdot v_{f}\cdot \tan\varphi \cdot \left(D-d\right)\cdot H_{v}}{2n_{0}}$$

where *v*_f_ is the workpiece feed rate (mm/s), *n*_0_ is the rotation speed of the trepanning cutter (r/min), *D* is the outer diameter of the trepanning cutter (mm), and *d* is the inner diameter of the trepanning cutter (mm).

The normal force *F*_an_ of all single abrasive particles participating in effective grinding is combined to form the axial force *F*_n_ of the cutter. Therefore, the normal force Fan of a single abrasive particle is expressed as follows:

(3) $$F_{n}=mF_{\mathrm{an}}$$

where *m* is the effective number of abrasive particles involved in machining.

Combining Equations (1)–(3) obtains the lateral crack length *C_l_* with process parameters:

(4) $$C_{l}={\left(\frac{15\pi }{2}\right)}^{5/8}\alpha {\left(\tan\varphi \right)}^{5/24}\frac{E^{3/8}H_{v}^{1/8}{\left(D-d\right)}^{5/8}v_{f}{}^{5/8}}{K_{\mathrm{IC}}^{1/2}m^{5/8}n_{0}{}^{5/8}}$$

According to the definition of the ten-point height *R*_z_ of micro unevenness, and the geometric relationship shown in Figure 2, it can be seen that the maximum distance between the peak and valley depth on the micro-profile of the measured workpiece surface is the length of the lateral crack generated by the outermost abrasive particles on the cutter end face, which is expressed as follows:

(5) $$R_{z}=C_{l}$$

According to the corresponding conversion relationship between the arithmetic mean height *S*_a_ and *R*_z_ on the surface, when *R*_z_ < 200 μm, there is an approximate relationship *S*_a_ ≈ *R*_z_/4. Therefore, the arithmetic average height *S*_a_ of the surface roughness of the hole wall generated by trepanning machining of hard and brittle materials is expressed as follows:

(6) $$S_{a}=\frac{1}{4}R_{z}$$

Eventually, combining Equations (4)–(6), the surface roughness of the hole wall *S*_a_ can be expressed as follows:

(7) $$S_{a}=\frac{1}{4}{\left(\frac{15\pi }{2}\right)}^{5/8}\alpha {\left(\tan\varphi \right)}^{5/24}\frac{E^{3/8}H_{v}^{1/8}{\left(D-d\right)}^{5/8}v_{f}{}^{5/8}}{K_{\mathrm{IC}}^{1/2}m^{5/8}n_{0}{}^{5/8}}$$

### 2.2. The Formation Mechanism of Exit-Chipping

During the drilling machining of hard and brittle materials, a chipping situation occurs at both the entry and exit points, but the formation mechanism and manifestation of entry-chipping and exit-chipping are different. When the axial force of the abrasive particles acts on the surface of the workpiece, the lateral crack propagates towards the free surface of the workpiece, forming material fracture. The entry-chipping is mainly caused by the removal of uniform small pieces of material chips. While exit-chipping refers to the unstable propagation of initial cracks inside the material under the axial force of abrasive particles, which is a macroscopic fracture phenomenon of brittle materials. Exit-chipping mainly involves the removal of a large area and strip-shaped material fractures, and its size is generally much larger than the size of the entry-chipping. The exit-chipping damage has a greater impact on the quality of the workpiece. Therefore, this article focuses on studying the exit-chipping mechanism and its impact on the quality of the workpiece.

**Figure 3:**
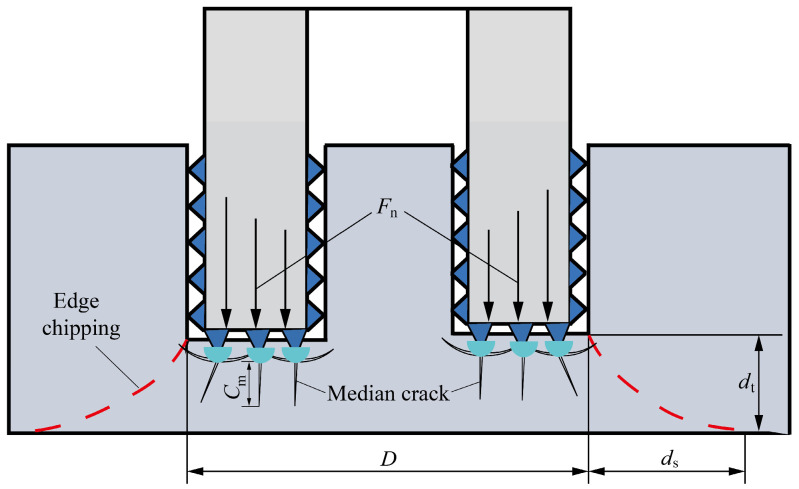
Mechanism of exit-chipping formation.

In the process of drilling hard and brittle materials, the diamond abrasive particles on the end face of the cutter press into the surface of the workpiece, generating radial cracks and lateral cracks inside the workpiece. The formation and propagation of lateral cracks cause the material to be removed from the surface in the form of brittle fracture, while the median cracks ultimately stay below the material surface, forming subsurface damage. As the cutting depth continues to increase, median cracks continuously form inside the workpiece, while the remaining material thickness *d*_t_ that bears the cutting force of the workpiece continuously decreases. When *d*_t_ is less than a certain critical value, the median crack becomes unstable and expands under the action of axial cutting force, resulting in exit-chipping damage. The formation mechanism of exit-chipping in hard and brittle materials is shown in Figure 3. From the figure, it can be seen that under a certain residual thickness at the bottom of the hole, the formation of exit-chipping is determined by the magnitude of the axial force of the cutter and the length of the median cracks on the subsurface. The exit-chipping width is determined by the remaining thickness of the hole bottom when the edge collapse is formed, and the exit-chipping width is proportional to the thickness of the edge collapse.

According to the theory of indentation fracture mechanics, the median crack depth generated by a single abrasive particle pressing into a brittle material *C*_m_ can be expressed as follows [15,16]:

(8) $$C_{m}=\alpha_{k}^{2/3}{\left(\frac{E}{H_{v}}\right)}^{1/3}{\left(\cot\varphi \right)}^{4/9}{\left(\frac{F_{\mathrm{an}}}{K_{\mathrm{IC}}}\right)}^{2/3}$$

where *α*_k_ is a dimensionless constant, taken as 0.042 [15].

Due to the rapid propagation of the median cracks, the remaining material suddenly loses stability under the axial force, resulting in an exit-chipping situation. According to the linear fracture mechanics of brittle materials, the conditions for the occurrence of exit-chipping are as follows [15]:

(9) $$\varepsilon F_{n}\sqrt{C_{m}}d_{s}^{-k}=K_{\mathrm{IC}}$$

where *ε* is a proportional constant independent of material properties, *d*_s_ is the exit-chipping width, and *k* is a dimensionless constant obtained through experiments.

Combining Equations (3), (8) and (9), the theoretical prediction model for the size of exit-chipping is expressed as follows:

(10) $$d_{s}^{k}=\varepsilon \alpha_{k}^{\frac{1}{3}}{\left(\cot\varphi \right)}^{\frac{2}{9}}K_{{}_{\mathrm{IC}}}^{-\frac{4}{3}}E^{\frac{1}{6}}H_{v}^{-\frac{1}{6}}m^{-\frac{1}{3}}F_{n}{}^{\frac{4}{3}}$$

We obtained experimental data on cutting force and exit-chipping size through machining K9 optical glass and adjusting the proportional constant *ε*. The theoretical prediction model for the size of exit-chipping obtained through data fitting is expressed as follows:

(11) $$d_{s}^{k}=\zeta K_{{}_{\mathrm{IC}}}^{-\frac{4}{3}}E^{\frac{1}{6}}H_{v}^{-\frac{1}{6}}m^{-\frac{1}{3}}F_{n}{}^{\frac{4}{3}}$$

where the magnitude of the two undetermined coefficients in the equation is: *k* = 1.6, *ζ* = 4.5 × 10^−3^ [15].

Eventually, combining Equations (2) and (11), the theoretical prediction model for the exit-chipping size that varies with process parameters can be obtained as follows:

(12) $$d_{s}^{1.6}=0.303{\left(\tan\varphi \right)}^{\frac{4}{3}}{\left(D-d\right)}^{\frac{4}{3}}K_{{}_{\mathrm{IC}}}^{-\frac{4}{3}}E^{\frac{1}{6}}H_{v}^{\frac{7}{6}}m^{-\frac{1}{3}}v_{f}{}^{\frac{4}{3}}n_{0}{}^{-\frac{4}{3}}$$

## 3. Results

### 3.1. Test Conditions and Methods

This experimental system is a deep-hole trepanning equipment developed and designed on the basis of the CW6163 ordinary lathe, with a maximum machining depth of 800 mm. The cutter used in the experiment is a fixed diamond deep-hole trepanning drill, with an outer diameter of 132 mm and an inner diameter of 124 mm. The wall thickness of the cutter arbor is 2 mm, and the granularity of the diamond abrasive particles is 70/80. The material of the workpiece is K9 optical glass, and the size of the workpiece is *Φ*150 mm × 20 mm. The cutting fluid used in the experimental system is water. Due to the fact that this experimental system is a specialized equipment independently designed on the basis of an ordinary lathe, there are certain limitations in adjusting the rotational speed and workpiece feed rate. At the same time, the diameter of the cutter rod of the trepanning drill is 130mm and the length is 935 mm, which leads to poor rigidity of the cutter. Therefore, the entire processing system is prone to unstable states under certain process parameters. After analyzing a large number of pre-experimental results, this article selected the process parameters in Table 2. The observation of the surface morphology characteristics of the hole wall and the measurement of the exit-chipping width are carried out using the VHX-5000 ultra depth of field three-dimensional microscopy system produced by KEYENCE Company in Osaka, Japan. The measurement of the surface roughness of the hole wall is carried out using the DCM3D white light confocal interference microscope produced by Leica Company in Wetzlar, Germany.

### 3.2. Surface Roughness Experiment Results

This article observes the surface morphology characteristics and measures the surface roughness of the workpiece processed under the above process parameters, as shown in Table 3.

From Table 3, it can be seen that under cutting conditions with a rotational speed of 90.5 rpm, as the feed rate continues to increase, the grooves on the surface of the workpiece gradually become sparse, but the depth of the grooves increases significantly, and the number of pits formed on the surface also increases significantly, and the corresponding surface roughness also gradually increases. Under cutting conditions with a workpiece feed rate of 20 μm/s, with the continuous increase in rotational speed, the density of grooves on the surface of the workpiece does not change significantly, but the depth of grooves decreases significantly. The pits formed on the surface also gradually decrease, and the corresponding surface roughness also gradually decreases. These changing trends demonstrate well that the surface roughness of the workpiece is mainly affected by the cutting force caused by changes in process parameters, and also prove that the surface roughness of the hole wall is mainly formed by the material detachment wrapped by transverse cracks.

### 3.3. Experimental Results of the Exit-Chipping Width

Table 4 shows the measurement results of the exit-chipping width of the workpiece processed under the above process parameters.

From the morphology of the exit-chipping in Table 4, it can be seen that the exit-chipping of K9 optical glass using a fixed abrasive trepanning cutter is mainly caused by large area and strip-shaped brittle material fracture. There are also a large number of small edge fractures on the inner side of the hole wall, and there is a clear positive correlation between the exit-chipping thickness and the exit-chipping width. Under cutting conditions with a rotational speed of 90.5 rpm, the exit-chipping width and thickness significantly increase with the continuous increase in feed rate. Under cutting conditions with a workpiece feed of 20 μm/s, with the continuous increase in rotational speed, the exit-chipping width and thickness significantly decrease.

## 4. Discussion

### 4.1. The Influence of Process Parameters on the Surface Roughness of Workpiece

In order to analyze the influence of process parameters on the surface roughness of workpieces and verify the consistency between the theoretical model of surface roughness of workpieces and the actual machining situation, a comparative analysis was conducted between the experimentally measured roughness values and the theoretical calculation values in Table 3. The experimental and theoretical values of the surface roughness of the workpiece under different process parameters are shown in Table 5.

The results from Table 5 show that the trend of the influence of the experimental process parameters on the surface roughness of the workpiece is completely consistent with the predicted trend of the model, indicating that the established surface roughness theoretical model can accurately predict the surface roughness of the workpiece as the process parameters change. The average error between the measured and predicted values of workpiece surface roughness is 13.15%, with a maximum error of 27.43%. The reason for the error in the surface roughness model of the workpiece may be due to the secondary grinding of the hole wall surface by the grinding particles on the side of the cutter, which improves the surface condition of the hole wall and reduces the surface roughness. Trepanning machining is a typical fully sealed machining process, where the cutting fluid is filled with a large amount of chips and detached abrasive particles, resulting in an uneven distribution of grooves, pits, and powdery surfaces on the hole wall surface. When selecting different measurement positions, the surface roughness may fluctuate greatly, resulting in certain measurement errors. In addition, the lateral vibration of the trepanning cutter leads to an uneven machining contact surface, unstable cutting volume of a single abrasive particle, and fluctuations in cutting force, ultimately resulting in varying surface roughness across different sections.

The theoretical values of the surface roughness of the workpiece under different process parameters are greater than the experimental values, and the performance is more obvious at a low feed rate and high speed. This is mainly because during the trepanning machining, the abrasive particles on the side of the cutter grind and trim the surface of the hole wall to varying degrees, and the peak height of the workpiece surface is overall reduced, resulting in an overall improvement in the unevenness of the workpiece surface. However, theoretical calculations cannot incorporate this part of the error into the model, so the experimental values of surface roughness are smaller than the theoretical values. When machining a workpiece at a low feed rate or high rotational speed, the grinding cycle of the cutter side abrasive particles on the hole wall is longer, the grinding of the hole wall surface is more uniform, and the density of the surface grooves formed is greater, reducing the displacement difference between the surface peaks and valleys of the workpiece. The surface roughness of the workpiece is greatly improved, so the error between the experimental and theoretical values of surface roughness is greater.

**Figure 4:**
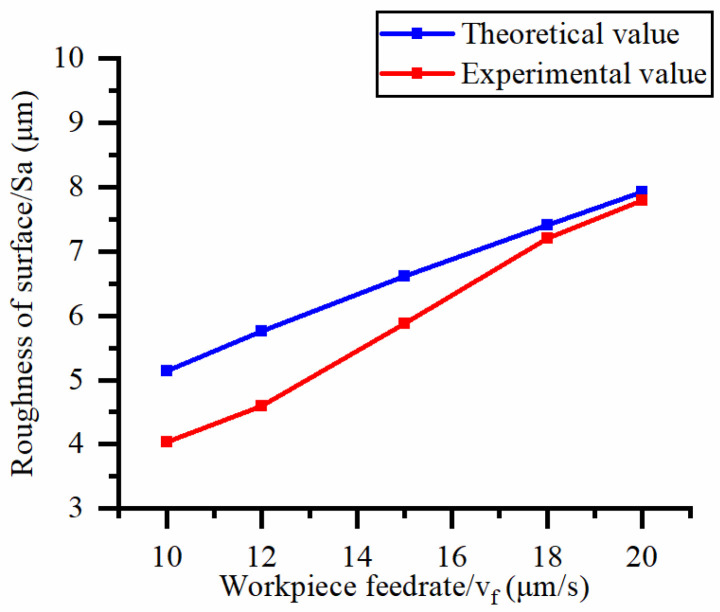
Effect of workpiece feed rate on surface roughness.

**Figure 5:**
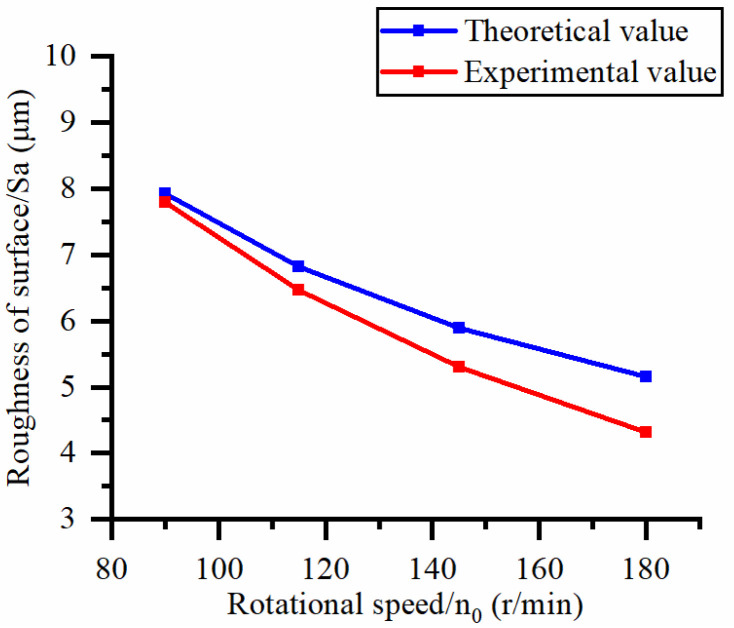
Effect of rotational speed on surface roughness.

This article uses the single-factor variable method to obtain the theoretical and experimental values of workpiece surface roughness under different process parameters. When the rotational speed is 90.5 rpm, the variation pattern of workpiece surface roughness with the change of workpiece feed rate is shown in Figure 4. The experimental and theoretical values of the surface roughness of the workpiece increase with the increase in the workpiece feed rate, and are close to a linear trend. When the feed rate of the workpiece is 20 μm/s, the variation pattern of the surface roughness of the workpiece with the change of rotational speed is shown in Figure 5. The experimental and theoretical values of the surface roughness of the workpiece decrease with the increase in the rotational speed, and there is good consistency in the trend between the experimental and theoretical values.

As the feed rate of the workpiece increases, the axial force of the abrasive grains on the end face of the trepanning cutter increases, causing the lateral cracks generated by the abrasive grains to extend deeper into the hole wall, resulting in larger and deeper pits formed by the material detachment on the hole wall surface. At the same time, the grinding cycle of the spiral moving cutter side abrasive grains on the hole wall decreases, resulting in a decrease in the density of the surface grooves and a gradual increase in surface roughness. And as the rotational speed increases, the actual cutting amount of the abrasive particles on the end face of the trepanning cutter decreases, and the axial force formed by the abrasive particles also decreases, resulting in a shallower depth of lateral cracks generated by the abrasive particles extending into the hole wall, reduced material detachment on the hole wall surface, and a smaller and shallower overall surface depression. At the same time, the grinding cycle of the cutter side abrasive particles on the hole wall increases, resulting in an increase in the density of surface grooves and a gradual decrease in surface roughness.

### 4.2. The Influence of Process Parameters on the Exit-Chipping Width

In order to analyze the influence of process parameters on the exit-chipping width and verify the consistency between the theoretical model of the exit-chipping width and the actual machining process, a comparative analysis was conducted between the experimentally measured exit-chipping width and the theoretical value in Table 4. The experimental and theoretical values of the exit-chipping width under different process parameters are shown in Table 6.

The results from Table 6 show that the trend of the influence in the process parameters obtained from the experiment on the exit-chipping width is completely consistent with the predicted trend of the model, indicating that the established theoretical model can accurately predict the exit-chipping width as the process parameters change. The average error between the measured and predicted values of the exit-chipping width is 6.73%, with a maximum error of 16.10%. The reason for the error in the exit-chipping width model may be the poor rigidity of the trepanning cutter, which causes fluctuations in axial force and affects the exit-chipping thickness, resulting in a corresponding change in the exit-chipping width. The uneven abrasive particles on the end face of the cutter lead to an uneven formation of median crack damage at the bottom of the hole, resulting in a certain error in the exit-chipping thickness throughout the entire hole. In addition, during the installation process of the workpiece, the end face is not perpendicular to the axial direction of the cutter, or the lateral vibration of the cutter causes the remaining thickness of the hole bottom material to be uneven. The parts with a smaller thickness of the hole bottom material reach the fracture strength of the material and begin to form local chipping. At this time, the cutting force borne by the parts with a larger thickness of the hole bottom material and no chipping suddenly increases, forming a larger exit-chipping width.

**Figure 6:**
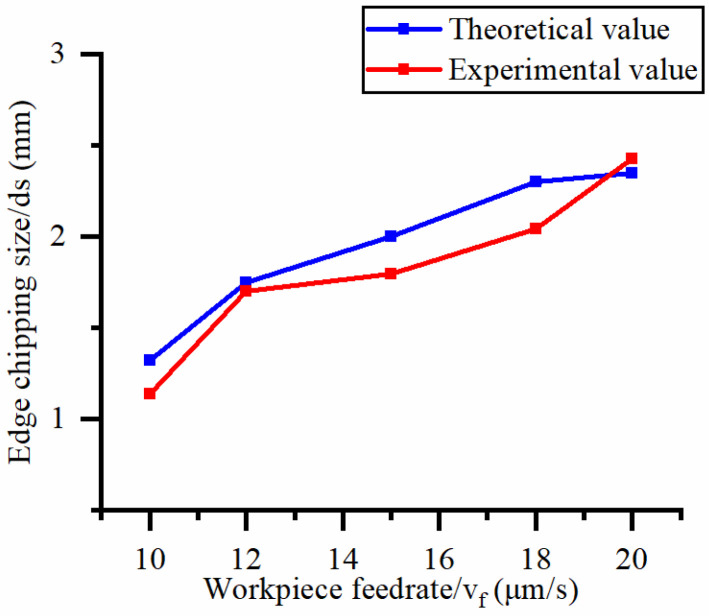
Influence of workpiece feed speed on the edge chipping size.

**Figure 7:**
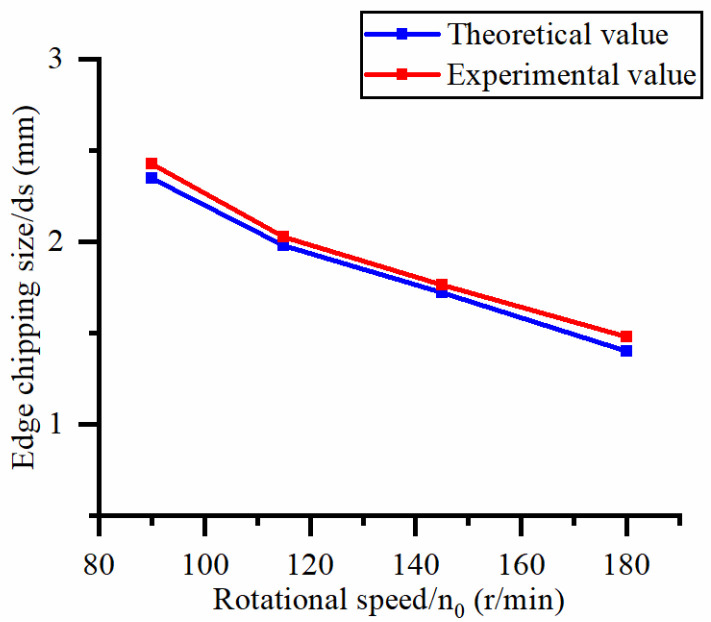
Influence of rotational speed on edge chipping size.

The single-factor variable method is used to obtain the theoretical and experimental values of the exit-chipping width of different process parameters. When the rotational speed is 90.5 rpm, the variation of the exit-chipping width with the change of workpiece feed rate is shown in Figure 6. In Figure 6, the experimental and theoretical values of the exit-chipping width increase with the increase in the workpiece feed rate. When the feed rate of the workpiece is 20 μm/s, the variation of the exit-chipping width with the rotational speed is shown in Figure 7. In Figure 7, the experimental and theoretical values of the exit-chipping width decrease with the increase in the rotational speed, and the trend of change between the experimental and theoretical values is consistent.

As the feed rate of the workpiece increases, the axial force of the abrasive grains on the end face of the trepanning cutter increases. At the same time, the median crack generated by the diamond abrasive grains inside the workpiece is deeper, causing more serious damage to the remaining material during machining. The remaining material exhibits an unstable fracture at a larger thickness, resulting in a larger exit-chipping width. As the rotational speed increases, the actual cutting amount of the abrasive particles on the end face of the trepanning cutter decreases, the axial force formed by the abrasive particles decreases, the median cracks generated inside the workpiece becomes shallower, the remaining material of the workpiece bears less axial force, and the surface damage also decreases. The thickness of the unstable fracture of the remaining material at the bottom of the hole decreases, and the formed exit-chipping width also decreases. Therefore, a lower feed rate of a workpiece and higher rotational speed machining can significantly improve the exit-chipping situation.

## 5. Conclusions

This article analyzes the formation mechanism of surface roughness and exit-chipping width during the drilling process of hard and brittle materials and establishes a mathematical prediction model for the surface roughness and exit-chipping width of hard and brittle materials processed with the fixed abrasive trepanning cutter. Drilling K9 optical glass test shows that the surface roughness of the workpiece and the exit-chipping width increase with the increase in feed rate and decrease with the increase in rotational speed. The experimental results have verified the accuracy of the established mathematical prediction model. The average errors of workpiece surface roughness and exit-chipping width are 13.15% and 6.73%, respectively. The main reasons for surface roughness errors are the secondary grinding of the hole wall surface by the abrasive particles on the side of the cutter, the large fluctuation of surface roughness, and the lateral vibration of the cutter. The fluctuation of axial force and inaccurate installation of the workpiece are the main reasons for the error in the exit-chipping width. This model can accurately predict the surface roughness and exit-chipping width of hard and brittle materials such as silicon, ceramics, and silicon carbide processed by fixed abrasive trepanning drilling. At the same time, the required surface roughness and exit-chipping width can be obtained by adjusting process parameters, providing some potential benefits for the widespread application of hard and brittle materials.

## Figures and Tables

**Table 1:** Previous research on trepanning processing of hard and brittle materials.

S.No.	Topics	Year	Author (s)	Summary of Work	Output
1	RUM and CD experiments	2014	Ding et al. [8]	The effects of RUM and CD on axial force, torque, hole quality, and drilling surface roughness.	The surface roughness of drilling holes obtained using RUM is lower than CD
2	Constant feed rate drilling experiments	2019	Zheng et al. [9]	Constant feed rate drilling experiments on Al_2_O_3_ and SiC materials using a diamond drill	The changes in axial force and hole wall surface microstructure during the drilling process
3	Experiments on drilling Al_2_O_3_ armored ceramics	2011	Gao et al. [10]	The effects of axial force and rotational speed on drilling efficiency and hole quality	Increasing axial force and rotational speed can improve drilling efficiency, but excessive axial force and excessive rotational speed can also reduce drilling efficiency.
4	Experiments on drilling ceramic composite armor	2012	Zheng et al. [11]	The effects of process parameters such as rotational speed and axial force on machining efficiency and hole wall surface quality	Choosing reasonable process parameters can greatly improve drilling efficiency
5	Experiments on rotary ultrasonic constant feed rate drilling of hard and brittle materials	2005	Li et al. [12,13]	The effects of parameters such as spindle speed, feed rate, and ultrasonic power on cutting force and size of hole edge collapse after processing	Spindle speed and feed rate, as well as their interaction, have significant effects on hole quality.
6	Experiments on soda glass using a fixed diamond abrasive cutter	2018	Abdelkawy et al. [14]	The effects of cutter surface abrasive density, rotational speed, and workpiece feeding on axial force and hole edge collapse	Low rotational speed and high feed rate would reduce the width of hole edge collapse
7	Experiments on drilling optical glass	2021	Sharma et al. [5]	The effects of drilling force and cutting temperature on the surface integrity of holes by drilling optical glass	Reducing cutting force and drilling temperature can reduce the stress during drilling, thereby improving the surface integrity of the workpiece
8	Formation mechanism of exit-chipping	2020	Lv et al. [6]	The formation mechanism of exit-chipping during RUD and CD of BK7 glass from both theoretical and experimental perspectives	A quantitative relationship between instantaneous extrusion pressure and crack propagation direction that is inversely proportional
9	Establishment of the prediction model for the hole-chipping size	2016	Wang et al. [15]	The influence of cutting force and machining-induced subsurface crack damage on hole-chipping.	A prediction model for the hole-chipping size during rotary ultrasonic drilling of hard and brittle materials

**Table 2:** Experimental parameters.

Serial Number	1	2	3	4	5	6	7	8
Rotational speed *n*_0_/(r/min)	90.5	90.5	90.5	90.5	90.5	113.5	144	181
Feed rate *v*_f_/(μm/s)	10	12	15	18	20	20	20	20

**Table 3:** Surface morphology characteristics and surface roughness of workpiece.

Process Parameters	Surface Morphology	Surface Roughness
No. 1 *n*_0_ = 90.5 rpm *v*_f_ = 10 μm/s	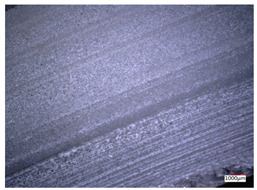	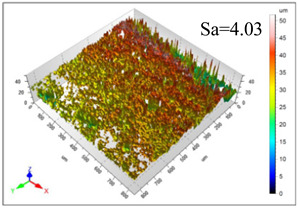
No. 2 *n*_0_ = 90.5 rpm *v*_f_ = 12 μm/s	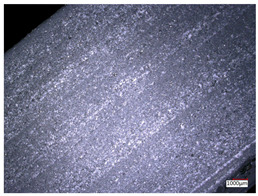	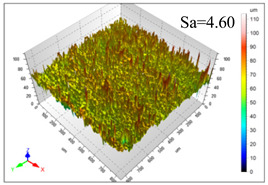
No. 3 *n*_0_ = 90.5 rpm *v*_f_ = 15 μm/s	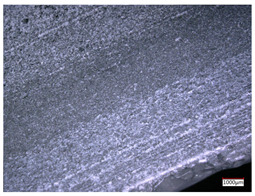	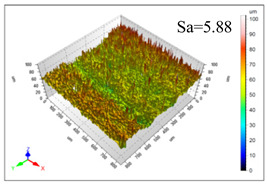
No. 4 *n*_0_ = 90.5 rpm *v*_f_ = 18 μm/s	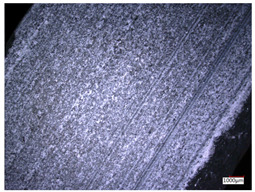	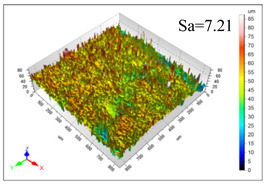
No. 5 *n*_0_ = 90.5 rpm *v*_f_ = 20 μm/s	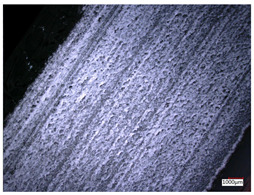	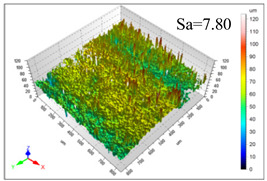
No. 6 *n*_0_ = 113.5 rpm *v*_f_ = 20 μm/s	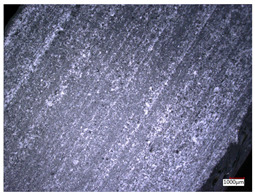	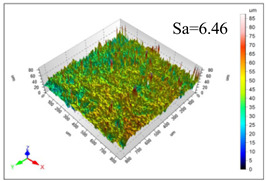
No. 7 *n*_0_ = 144 rpm *v*_f_ = 20 μm/s	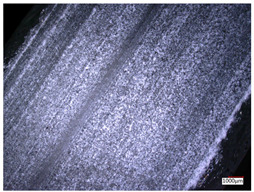	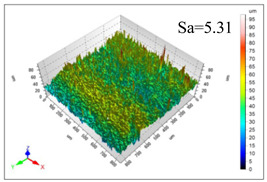
No. 8 *n*_0_ = 181 rpm *v*_f_ = 20 μm/s	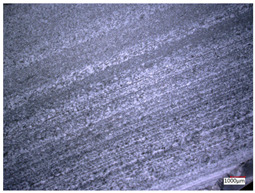	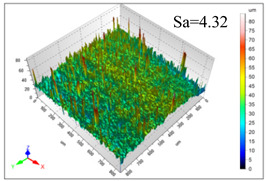

**Table 4:** Characteristics of exit-chipping on workpiece under different process parameters.

Process parameters	No. 1 *n*_0_ = 90.5 rpm; *v*_f_ = 10 μm/s *d*_s_ = 1.14	No. 2 *n*_0_ = 90.5 rpm; *v*_f_ = 12 μm/s *d*_s_ = 1.70
Exit-chipping image	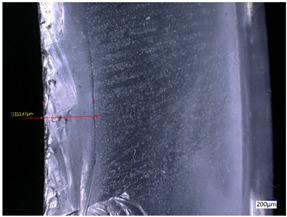	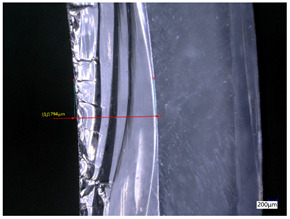
Process parameters	No. 3 *n*_0_ = 90.5 rpm; *v*_f_ = 15 μm/s *d*_s_ = 1.79	No. 4 *n*_0_ = 90.5 rpm; *v*_f_ = 18 μm/s *d*_s_ = 2.04
Exit-chipping image	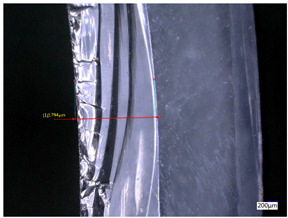	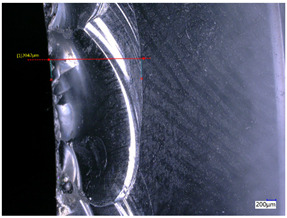
Process parameters	No. 5 *n*_0_ = 90.5 rpm; *v*_f_ = 20 μm/s *d*_s_ = 2.43	No. 6 *n*_0_ = 113.5 rpm; *v*_f_ = 20 μm/s *d*_s_ = 2.03
Exit-chipping image	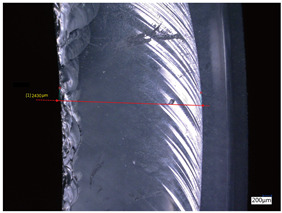	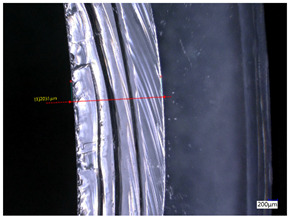
Process parameters	No. 7 *n*_0_ = 144 rpm; *v*_f_ = 20 μm/s *d*_s_ = 1.76	No. 8 *n*_0_ = 181 rpm; *v*_f_ = 20 μm/s *d*_s_ = 1.48
Exit-chipping image	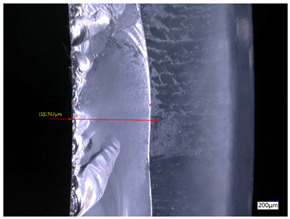	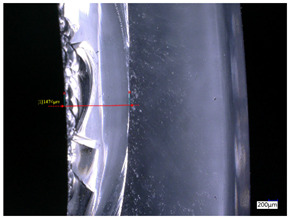

**Table 5:** Comparison between experimental and theoretical calculation results of workpiece surface roughness.

Serial Number	Rotational Speed *n*_0_/(r/min)	Feed Rate *v*_f_/(μm/s)	Theoretical Values *S*_a_/(μm)	Experimental Values *S*_a_/(μm)	Error (%)
1	90.5	10	5.14	4.03	27.43
2	90.5	12	5.76	4.60	25.11
3	90.5	15	6.62	5.88	12.52
4	90.5	18	7.41	7.21	2.84
5	90.5	20	7.91	7.80	1.53
6	113.5	20	6.82	6.46	5.55
7	144	20	5.90	5.31	11.08
8	181	20	5.15	4.32	19.28

**Table 6:** Comparison between experimental and theoretical values of the exit-chipping width.

Serial Number	Rotational Speed *n*_0_/(r/min)	Feed Rate *v*_f_/(μm/s)	Theoretical Values *d*_s_/(mm)	Experimental Values *d*_s_/(mm)	Error (%)
1	90.5	10	1.32	1.14	16.10
2	90.5	12	1.75	1.70	2.94
3	90.5	15	1.95	1.79	8.70
4	90.5	18	2.30	2.04	12.63
5	90.5	20	2.35	2.43	−3.21
6	113.5	20	1.98	2.03	−2.46
7	144	20	1.72	1.76	−2.38
8	181	20	1.40	1.48	−5.41

## Data Availability

Not applicable.

## Nomenclature

*C_l_*	Lateral crack length (μm)
*α*	Dimensionless constant
*φ*	Half angle of the abrasive tip (°)
*E*	Elastic modulus of the material (GPa)
*K*_IC_	Fracture toughness of the material (MPa·m^1/2^)
*H*_v_	Hardness of the material (GPa)
*F*_an_	Axial force exerted on a single abrasive particle (N)
*F*_n_	Axial force exerted on the cutter (N)
*v*_f_	Workpiece feed rate (μm/s)
*n*_0_	Rotation speed of trepanning cutter (r/min)
*D*	Outer diameter of the trepanning cutter (mm)
*d*	Inner diameter of the trepanning cutter (mm)
*m*	Effective number of abrasive particles involved in machining
*R*_z_	Maximum distance between the peak and valley depth on the micro profile of the measured workpiece surface (μm)
*S*_a_	Surface roughness of the hole wall (μm)
*C*_m_	Median crack depth generated by a single abrasive particle pressing into a brittle material (μm)
*ε*	Proportional constant independent of material properties
*d*_s_	Exit-chipping width (mm)
